# Ulcerative jejunitis in a child with celiac disease

**DOI:** 10.1186/1471-230X-14-29

**Published:** 2014-02-13

**Authors:** Terry Sigman, Van-Hung Nguyen, Florin Costea, Ana Sant’Anna, Ernest G Seidman

**Affiliations:** 1 Division of Pediatric Gastroenterology, Montreal Children’s Hospital, Faculty of Medicine, McGill University, 2300 Tupper St, Montreal, Quebec H3H 1P3, Canada; 2 Department of Pathology, Montreal Children’s Hospital, Faculty of Medicine, McGill University, 2300 Tupper St, Montreal, Quebec H3H 1P3, Canada

**Keywords:** Ulcerative jejunitis, Celiac disease, Pediatric, Capsule endoscopy

## Abstract

**Background:**

Celiac disease can present in children and adults with a variety of manifestations including a rare complication known as ulcerative jejunitis. The latter has been associated with refractory celiac disease in adult onset patients. The objective of this case report is to describe the first pediatric case of ulcerative jejunitis in celiac disease, diagnosed by capsule endoscopy, which was not associated with refractory celiac disease.

**Case presentation:**

The 9 year old girl presented with a history of abdominal pain and vomiting. Laboratory investigations revealed a slightly elevated IgA tissue transglutaminase antibody level in the setting of serum IgA deficiency. Initial upper endoscopy with biopsies was not conclusive for celiac disease. Further investigations included positive IgA anti-endomysium antibody, and positive HLA DQ2 typing. Video capsule endoscopy showed delayed appearance of villi until the proximal to mid jejunum and jejunal mucosal ulcerations. Push enteroscopy with biopsies subsequently confirmed the diagnosis of celiac disease and ulcerative jejunitis. Immunohistochemical studies of the intraepithelial lymphocytes and PCR amplification revealed surface expression of CD3 and CD8 and oligoclonal T cell populations. A repeat capsule study and upper endoscopy, 1 year and 4 years following a strict gluten free diet showed endoscopic and histological normalization of the small bowel.

**Conclusion:**

Ulcerative jejunitis in association with celiac disease has never previously been described in children. Capsule endoscopy was essential to both the diagnosis of celiac disease and its associated ulcerative jejunitis. The repeat capsule endoscopy findings, one year following institution of a gluten free diet, also suggest that ulcerative jejunitis is not always associated with refractory celiac disease and does not necessarily dictate a poor outcome.

## Background

Celiac disease (CD), or gluten-sensitive enteropathy, is an autoimmune disorder in which genetically susceptible individuals develop inflammatory changes in the small intestinal mucosa following the ingestion of gluten, a protein found in grains including wheat, rye and barley [1]. Clinical presentations vary extensively, from patients who are asymptomatic to those having one or more gastrointestinal symptoms such as abdominal pain, vomiting, diarrhea and bloating. Extraintestinal manifestations may include malnutrition and growth delay in children, osteoporosis, infertility, neurological symptoms and arthritis. Ulcerative jejunitis is a rare complication of CD typically found in adults in the 5th or 6th decade. It is usually associated with refractory celiac disease (RCD) and the possible development of enteropathy-associated T-cell lymphoma (EATL) [2,3]. Serological tests accurately identify those for whom a small bowel biopsy is indicated. The most specific serological tests available include IgA anti-endomysium (EMA) and IgA anti-tissue transglutaminase (tTG). The characteristic changes seen on small bowel biopsy include various degrees of villous atrophy with increased intraepithelial lymphocytes (IEL) and crypt hyperplasia, as summarized in the modified Marsh-Oberhuber Classification criteria [4]. The classic criteria for diagnosis of CD in a child with symptoms suggestive of CD include the characteristic histological changes on small bowel biopsy and clinical resolution after starting a gluten free diet (GFD) [1]. Positive serological tests that revert to normal following a GFD are supportive of the diagnosis [1]. Wireless capsule endoscopy of the small bowel (CE) is occasionally used as an adjunctive diagnostic test and has been shown to be helpful for detecting celiac disease [5]. CE provides high-resolution magnified views of the small intestinal mucosa and mucosal abnormalities include scalloping of folds, fissures or grooves, a mosaic pattern and absence or reduced duodenal folds [6].

In this case report we present the case of a child with ulcerative jejunitis associated with celiac disease that was diagnosed with CE and whose clinical symptoms, histological and capsule findings eventually resolved on a GFD.

## Case presentation

A 9-year-old girl was referred for assessment of a 1 year history of episodic abdominal pain and non-bilious vomiting. The pain was described as dull, localized to the periumbilical area and unassociated with food. A breath test was consistent with lactose malabsorption. However, lactose restriction only transiently improved symptoms. The review of systems and past medical history were unremarkable. She denied the use medications including nonsteroidal anti-inflammatory drugs (NSAIDS). There was no family history of celiac disease, pancreatitis, inflammatory bowel disease, or autoimmune disorders other than multiple sclerosis (paternal grandmother). Her physical exam was within normal limits with satisfactory weight and growth parameters.

Initial laboratory investigations revealed a normal hemoglobin and white blood cell count, erythrocyte sedimentation rate and serum albumin. Her tTG was discretely elevated (10 U/ml; normal < 5). However, her total serum IgA was low (<0.06 g/L; normal 0.63-3.49), whereas her IgE was elevated (986 ug/L; normal <240).

An initial upper endoscopy revealed small apthous ulcerations in the duodenal bulb. The second portion of the duodenum appeared normal. One duodenal biopsy revealed mild villous blunting and acute duodenitis, without an increased IEL count. No giardia or other parasites were seen. Two other duodenal biopsies revealed normal villi, with moderate eosinophlic infiltration involving the glands (40–50 eosinophils per high power field (HPF)). The pathology report concluded that the findings were insufficient for a diagnosis of celiac disease and that the focal eosinophilic infiltrates were consistent with infectious, allergic or eosinophilic gastroenteropathies. A colonoscopy revealed mild inflammatory changes in the terminal ileum and ileocecal valve as well as hyperemia of the mucosa in the rectosigmoid area. Histological analysis showed focal active ileitis, and foci of mild active colitis with intraepithelial eosinophils. No granulomas, crypt abscesses, or chronic inflammatory changes were seen. She was given a tentative diagnosis of eosinophilic gastroenteritis and was treated with once daily oral montelukast sodium. No sustained benefit was achieved.

**Figure 1:**
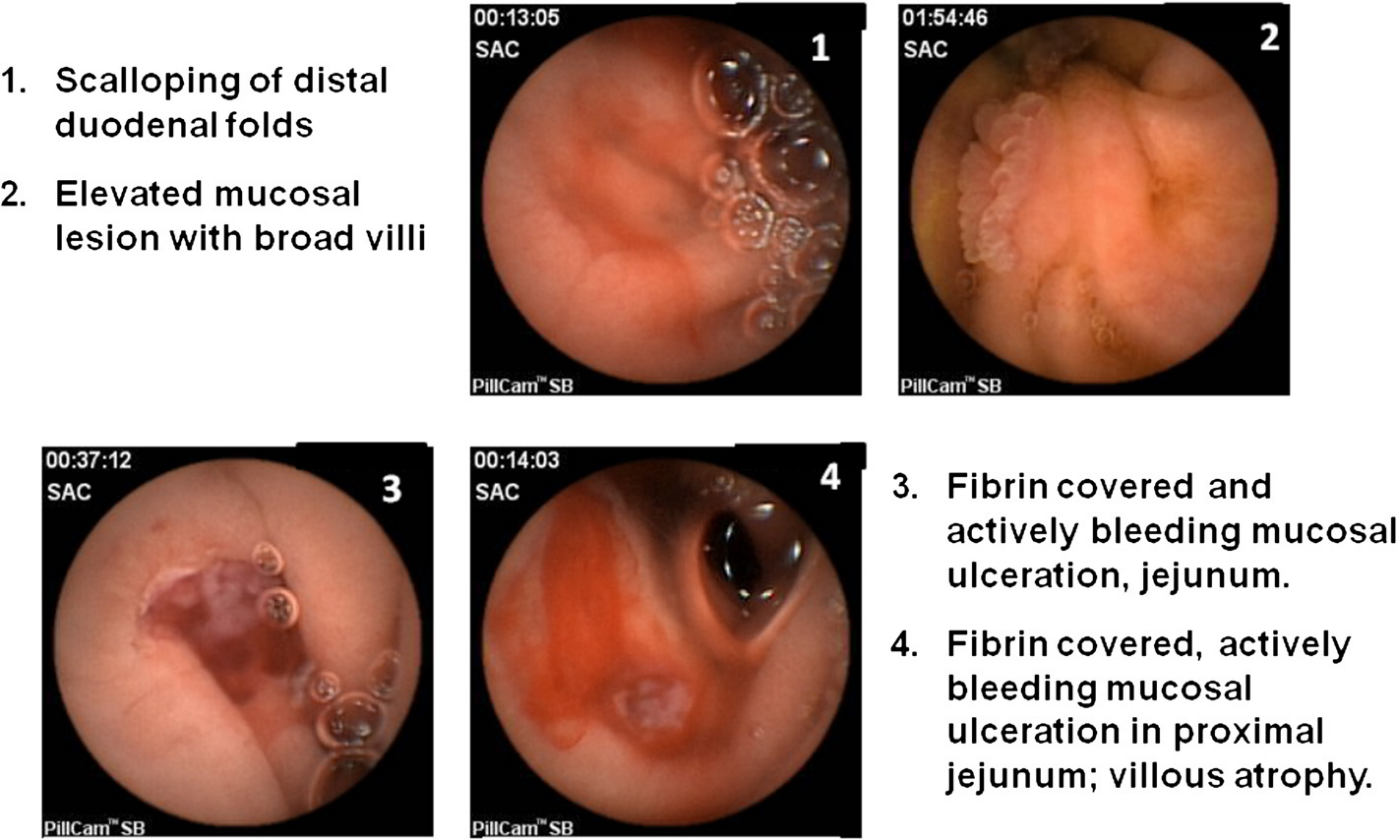
**WIRELESS CAPSULE ENDOSCOPY Initial Exam (Normal Diet).** Findings highly suggestive of celiac disease complicated by ulcerative jejunitis. Push enteroscopy suggested to obtain diagnosis and to rule out EATL.

**Figure 2:**
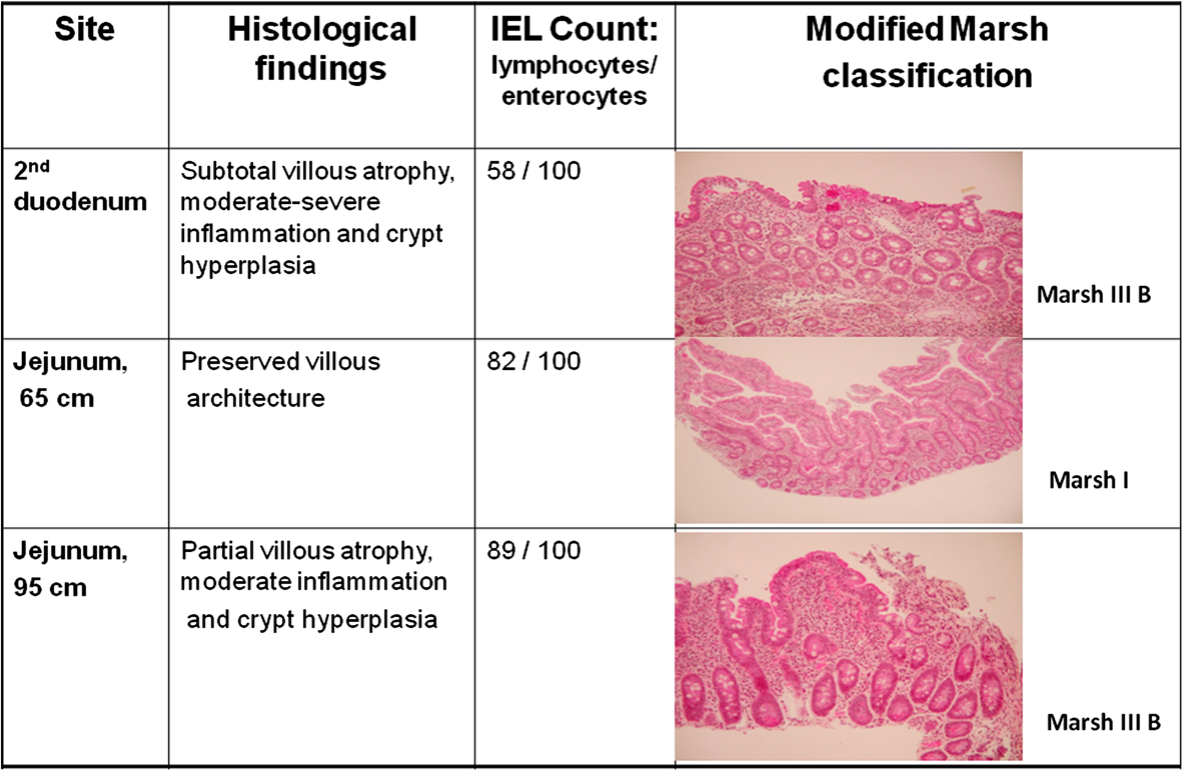
**Histological findings on a normal diet.** Histological confirmation of celiac disease by push enteroscopy.

In view of persistent symptoms, further investigations were pursued, which revealed positive EMA and HLA DQ2 typing. CE of the small bowel showed patchy villous atrophy with delayed appearance of villi until the proximal to mid jejunum, as well as areas with slight mucosal scalloping. Moreover, fibrin covered actively bleeding mucosal ulcerations were observed in the jejunum (Figure 1). Her tTG remained slightly elevated (7 U/ml; normal < 5). Push enteroscopy performed 7 months following her initial upper endoscopy, confirmed ulcerative jejunitis. Biopsies revealed subtotal villous atrophy, crypt hyperplasia and an IEL count of 58 lymphocytes/100 enterocytes in the second stage of the duodenum (Marsh classification III B). Changes in the jejunum varied from preserved villous architecture with 82 IEL/100 enterocytes (Marsh I at 65 cm) to partial villous atrophy and crypt hyperplasia with an IEL count of 89/100 enterocytes (Marsh III B at 95 cm), consistent with a diagnosis of celiac disease (Figure 2).

**Figure 3:**
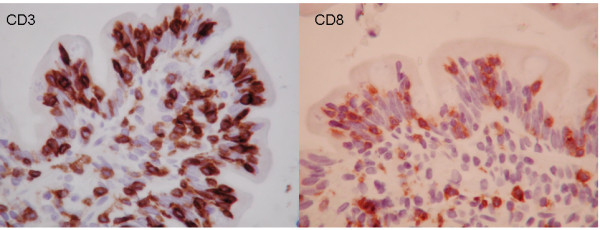
**Immunohistochemical phenotyping of IELs (at 95 cm in jejunum) at time of diagnosis.** Intraepithelial lymphocytosis (IEL), 89 lymphocytes/100 enterocytes with double positivity for CD3 (LEFT) and CD8 (RIGHT), and negative for CD4; consistent with Marsh III A classification.

**Figure 4:**
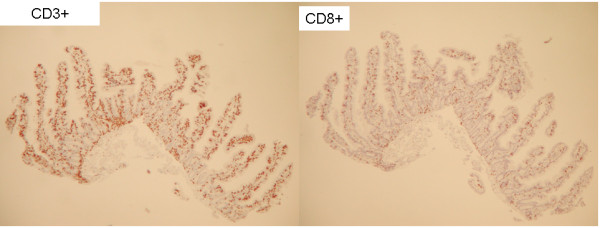
**Histology and immunohistochemical phenotyping of IELs (at 150 cm in jejunum) 6 months after starting GFD.** Intraepithelial lymphocytosis (IEL), 40 lymphocytes/100 enterocytes with double positivity for CD3 (LEFT) and CD8 (RIGHT), and negative for CD4; consistent with Marsh I classification.

Institution of a strict GFD for 6 months led to clinical improvement, with considerably less pain and cessation of vomiting. Moreover, her serum tTG normalized (<3 U/ml). However, a repeat CE again revealed delayed appearance of villi until the mid jejunum and mucosal ulcerations in the proximal jejunum without active bleeding, consistent with persistent ulcerative jejunitis. Biopsies obtained by repeat push enteroscopy after 6 months on a GFD showed normal villous architecture in the duodenum and jejunum down to 150 cm, with an IEL count of 12/100 enterocytes per HPF in the duodenum (Marsh 0) and 40-46/100 per HPF in the jejunum at 130 cm and 150 cm respectively (Marsh I). The biopsies were subsequently sent for lymphocytic identification and PCR amplification studies for T cell rearrangement on fresh tissue. PCR amplification studies were also done on paraffin embedded tissue from the samples at diagnosis. On the sample from initial diagnosis prior to GFD, a T-cell clonal population was observed although it stained positive for surface expression of both CD3 and CD8 (Figure 3). The specimen obtained after 6 months on a GFD showed oligoclonal T-cell populations and still stained positive for surface expression of both CD3 and CD8 (Figure 4). Since there was definite improvement histologically, a decision was made to continue the strict GFD and to re-evaluate by CE and histology after another 6 months.

A third CE after 12 months on a GFD was unremarkable, with early appearance of normal villi in the duodenum and no evidence of ulcerative jejunitis. A duodenal biopsy now showed well preserved villous morphology and a normal IEL count (25 lymphocytes/100 enterocytes; CD3/CD8 positive), consistent with celiac disease in remission. A recent capsule, done 4 years after diagnosis, was unremarkable and multiple duodenal biopsies were completely normal as well. Her tTG remained normal (<3 U/ml), her EMA normalized and her total serum IgA remained low (0.4gL; normal 0.82-4.57).

## Discussion

Most cases of CD are easily diagnosed with routine serological tests and duodenal biopsies. Unfortunately however, the diagnosis of celiac disease is not always straight forward. Standard serological testing does not always identify the need for a small intestinal biopsy because CD occurs with a higher frequency in individuals with selective IgA deficiency, as in the present case [7]. A small percentage of patients with CD are seronegative [8]. Moreover, not all cases of CD have the classic histological criteria. Histological findings can vary due to patchy distribution or varying degrees of villous atrophy [4]. Furthermore, intraepithelial lymphocytosis, crypt hyperplasia and villous atrophy can be seen in other conditions affecting the small bowel, such as giardiasis, Crohn’s disease, autoimmune and allergic enteropathies [1,4,9]. A diagnostic dilemma arose in this case in that the serology was borderline positive as the patient is IgA deficient. Additionally, the initial histopathological findings were not conclusive for CD. We thus proceeded with CE which revealed ulcerative jejunitis in addition to typical changes of CD. This is consistent with the recent study [10] showing that CE is able to accurately diagnose villous atrophy in CD as well as complications such as ulcerative jejunitis.

Ulcerative jejunitis is rare, but can be seen in infections, or inflammatory conditions such as Crohn’ disease or eosinophilic gastroenteritis, collagen vascular and other immune-mediated diseases such as vasculitis, systemic lupus erythematosis or Henoch-Schonlein purpura, as well as secondary to medications such as aspirin or other NSAIDS, RCD, traumatic conditions such as intussusception, immunodeficiency disorders such as hypogammaglobulinemia or various neoplastic conditions [11]. In the pediatric literature, 4 cases of ulcerative jejunitis were diagnosed by CE in young children suffering from juvenile rheumatoid arthritits, three of which were attributed to NSAID use [12].

Among adult cases, ulcerative jejunitis is usually associated with RCD [2,3]. The definitions of RCD vary in the literature. RCD can be defined as either a primary non-response to a GFD or a secondary form, where patients have a relapse of their enteropathy despite a GFD [13]. Generally, RCD is diagnosed if there is no histological response to a GFD for more than 12 months or if severe symptoms persist necessitating intervention independent of duration of the GFD [14,15]. In fact, it was reported that it may take up to two years for histological recovery in a patient with uncomplicated CD on a GFD [14]. RCD is classified as types 1 and 2, depending on the surface expression of intraepithelial T-cells [16]. In type I RCD, the IEL express CD3, CD4 or CD8, CD103 and T-cell receptors (TCR) with polyclonal TCR gene rearrangement. These phenotypically normal IEL are indistinguishable from those seen in active, uncomplicated CD. In type II RCD, IEL lack surface CD3, CD4 or CD8, and TCR, but express intracytoplasmatic CD3 and surface CD103. The aberrant IEL in type II RCD are often characterized by monoclonal rearrangement of the TCR on the genomic level [17,18]. Such aberrant IEL have also been reported in ulcerative jejunitis and EATL, conditions that may co-exist or evolve from RCD [19]. These observations suggest that these disorders constitute a spectrum with type II RCD. A pediatric case of type I RCD was recently reported [20]. Although the clinical course is generally benign, type I RCD is associated with an increased risk of developing autoimmune disorders. Patients not responding to a strict GFD may benefit from immunosuppressive drugs [21,22], including thioguanine [23]. Type II RCD is associated with a risk of lymphoma and thus potentially a poor prognosis. Although no treatment has been found to be curative, chemotherapeutic agents are generally administered [24]. In severe cases, autologous stem cell transplantation has been employed for enteropathy associated T cell lymphoma [25].

The patient described herein is, to the best of our knowledge, unique in presenting with ulcerative jejunitis in association with CD in the pediatric age group. Furthermore, the ulcerative jejunitis did not seem to be associated with RCD in this particular case. We did observe resolution of the ulcerative jejunitis as well as the enteropathy after 12 months on a GFD both histologically as well as shown by repeat CE. Moreover, despite having ulcerative jejunitis, she never had abnormal T-cell phenotypes or evidence of EATL. In cases with ulcerative jejunitis, with or without RCD, a phenotypical analysis of IELs should be performed by immunohistochemistry, or if possible, using flow cytometry in order to distinguish between the two subtypes [26].

## Conclusion

Ulcerative jejunitis in association with CD has never, based on our review of the literature, been described in the pediatric age group. CE was essential to establishing the initial diagnosis of CD in this patient and in identifying the associated ulcerative jejunitis, as well as its resolution. CE was also very helpful in guiding the need for tissue sampling and in following the outcome of ulcerative jejunitis complicating CD after institution of a GFD. The CE findings suggest that ulcerative jejunitis is not always associated with RCD, and as in this case, does not necessarily dictate a poor outcome. Further studies are needed to evaluate the incidence and outcome of ulcerative jejunitis and RCD in children with celiac disease.

## Consent

Written informed consent was obtained from the patient for publication of this case report and any accompanying images. A copy of the written consent is available for review by the Editor of this journal. As the patient is a minor, parental consent was also obtained.

## Competing interests

The authors declare that they have no competing interests.

## Authors’ contributions

TS: Prepared manuscript, initial primary gastroenterologist and endoscopist. VN: Pathologist implicated in reviewing the histology. FC: Reviewed video capsule endoscopy literature. AS: Read the follow up capsule studies. ES: Helped in preparation of manuscript, endoscopist, read the video capsule studies and current primary gastroenterologist. All authors read and approved the final manuscript.

## Pre-publication history

The pre-publication history for this paper can be accessed here:

http://www.biomedcentral.com/1471-230X/14/29/prepub

## References

[B1] Hill ID Dirks MH Liptak GS Colletti RB Fasano A Guandalini S Hoffenberg EJ Horvath K Murray JA Pivor M Seidman EG North American Society for Pediatric Gastroenterology, Hepatology and Nutrition Guideline for the diagnosis and treatment of celiac disease in children: recommendations of the North American society for pediatric gastroenterology, hepatology and nutrition J Pediatr Gastroenterol Nutr 2005 40 1 1 19 10.1097/00005176-200501000-00001 15625418

[B2] Elsing C Placke J Gross-Weege W Ulcerative jejunoileitis and enteropathy-associated T-cell lymphoma Eur J Gastroenterol Hepatol 2005 17 12 1401 1405 10.1097/00042737-200512000-00021 16292096

[B3] Biagi F Lorenzini P Corazza CR Literature review on the clinical relationship between ulcerative jejunoileitis, celiac disease, and enteropahty-associated T-cell lymphoma Scand J Gastroenterol 2000 35 8 785 790 10.1080/003655200750023129 10994614

[B4] Dickson BC Streutker CJ Chetty R Coeliac disease: an update for the pathologist J Clin Pathol 2006 59 1008 1016 10.1136/jcp.2005.035345 17021129 PMC1861744

[B5] Green PH Cellier C Celiac disease New Engl J Med 2007 357 1731 1743 10.1056/NEJMra071600 17960014

[B6] Cellier C Green PHR Collin P Murray J ICCE concensus for celiac disease Endocsopy 2005 37 10 1055 1059 10.1055/s-2005-870310 16189790

[B7] Catassi C Fabiani E Rätsch IM Coppa GV Giorgi PL Pierdomenico R Alessandrini S Iwanejko G Domenici R Mei E Miano A Marani M Bottaro G Spina M Dotti M Montanelli A Barbato M Viola F Lazzari R Vallini M Guariso G Plebani M Cataldo F Traverso G Ventura A The coeliac iceberg in Italy. A multicentre antigliadin antibodies screening for coeliac disease in school-age subjects Acta Paediatr Suppl 1996 412 29 35 8783752 10.1111/j.1651-2227.1996.tb14244.x

[B8] Mulder CJJ Cellier C Coeliac disease: changing views Best Pract Res Clin Gastroenterol 2005 19 3 313 321 10.1016/j.bpg.2005.01.006 15925838

[B9] Ho-Yen C Chang F van der Walt J Mitchell T Ciclitira P Recent advances in refractory coeliac disease: a review Histopathology 2009 54 7 783 795 10.1111/j.1365-2559.2008.03112.x 18700844

[B10] Barret M Malamut G Rahmi G Samaha E Edery J Verkarre V Macintyre E Lenain E Chatellier G Cerf-Bensussan N Cellier C Diagnostic yield of capsule endoscopy in refractory celiac disease Am J Gastroenterol 2012 107 10 1546 1553 10.1038/ajg.2012.199 22964554

[B11] Proctor D Panzini L Feldman M, Friedman L, Sleisenger M Isolated and diffuse ulcers of the small intestine Sleisenger & Fordtran’s Gastrointestinal and Liver Disease. Volume 2 2002 7 Philadelphia: Saunders 2081 2082

[B12] Fritscher-Ravens A Scherbakov P Bufler P Torroni F Ruuska T Nuutinen H Thomson M Tabbers M Milla P The feasibility of wireless capsule endoscopy in detecting small intestinal pathology in children under the age of 8 years: a multicenter European study Gut 2009 58 1467 1472 10.1136/gut.2009.177774 19625281

[B13] O’Mahony S Howdle PD Losowsky MS Review article: management of patients with non-responsive coeliac disease Aliment Pharmacol Ther 1996 10 5 671 680 10.1046/j.1365-2036.1996.66237000.x 8899074

[B14] Daum S Cellier C Mulder C Refractory coeliac disease Best Pract Res Clin Gastroenterol 2005 19 3 413 424 10.1016/j.bpg.2005.02.001 15925846

[B15] Tennyson CA Green PH The role of capsule endoscopy in patients with nonresponsive celiac disease Gastrointest Endosc 2011 74 6 1323 1324 10.1016/j.gie.2011.07.021 22136777

[B16] Wahab PJ Meijer JW Mulder CJ Histological follow-up of people with celiac disease on a gluten-free diet Am J Clin Pathol 2002 118 3 459 463 10.1309/EVXT-851X-WHLC-RLX9 12219789

[B17] Cellier C Delabesse E Helmer C Patey N Matuchansky C Jabri B Macintyre E Cerf-Bensussan N Brousse N Refractory sprue, coeliac disease, and enteropathy-associated T-cell lymphoma. French Coeliac Disease Study Group Lancet 2000 356 9225 203 208 10.1016/S0140-6736(00)02481-8 10963198

[B18] Cellier C Patey N Mauvieux L Jabri B Delabesse E Cervoni JP Burtin ML Guy-Grand D Bouhnik Y Modigliani R Barbier JP Macintyre E Brousse N Cerf-Bensussan N Abnormal intestinal intraepithelial lymphocytes in refractory sprue Gastroenterology 1998 114 3 471 481 10.1016/S0016-5085(98)70530-X 9496937

[B19] Bagdi E Diss TC Munson P Isaacson PG Mucosal intra-epithelial lymphocytes in enteropathy-associated T-cell lymphoma, ulcerative jejunitis, and refractory celiac disease constitute a neoplastic population Blood 1999 94 1 260 264 10381521

[B20] Daum S Weiss D Hummel M Ullrich R Heise W Stein H Riecken EO Foss HD Intestinal Lymphoma Study Group Frequency of clonal intraepithelial T lymphocyte proliferations in enteropathy-type intestinal T cell lymphoma, coeliac disease, and refractory sprue Gut 2001 49 6 804 812 10.1136/gut.49.6.804 11709515 PMC1728529

[B21] Mubarak A Oudshoorn JH Kneepkens CM Butler JC Schreurs MW Mulder CJ Houwen RH A child with refractory coeliac disease J Pediatr Gastroenterol Nutr 2011 53 2 216 218 10.1097/MPG.0b013e318214553a 21788766

[B22] Goerres MS Meijer JW Wahab PJ Kerckhaert JA Groenen PJ Van Krieken JH Mulder CJ Azathioprine and prednisone combination therapy in refractory coeliac disease Aliment Pharmacol Ther 2003 18 5 487 494 10.1046/j.1365-2036.2003.01687.x 12950421

[B23] Tack GJ van Asseldonk DP van Wanrooij RLJ van Bodegraven AA Mulder CJ Tioguanine in the treatment of refractory coeliac disease – a single centre experience Aliment Pharmacol Ther 2012 36 274 281 10.1111/j.1365-2036.2012.05154.x 22646133

[B24] Al-Toma A Verbeek WH Mulder CJ The management of complicated celiac disease Dig Dis 2007 25 3 230 236 10.1159/000103891 17827946

[B25] Jantunen E Boumendil A Finel H Luan J-J Rambaldi A Autologous stem cell transplantation for enteropathy-associated T-cell lymphoma: a retrospective study by the EBMT Blood 2013 121 13 2529 2532 10.1182/blood-2012-11-466839 23361910

[B26] Verbeek WH Goerres MS von Blomberg BM Oudejans JJ Scholten PE Hadithi M Al-Toma A Schreurs MW Mulder CJ Flow cytometric determination of aberrant intra-epithelial lymphocytes predicts T-cell lymphoma development more accurately than T-cell clonality analysis in refractory celiac disease Clin Immunol 2008 126 1 48 56 10.1016/j.clim.2007.09.002 18024205

